# 

**Published:** 1916-04

**Authors:** 


### Contents

Event and Comment ........................................ 151

Chronic Oral Infections and Their Relation to Diseases in Other Parts ........................................ 154

The Duty of the Dentist in Artificial Restoration, in Cases Where the Choice Lies Between Removable Bridge, Fixed Bridge, or Partial Plate ........................................ 169

Can I Be a Better Dentist ........................................ 175

Dentists in the Army and Navy ........................................ 183

Indents ........................................ 188

Announcements ........................................ 200

